# Cardiovascular autonomic effects of electronic cigarette use: a systematic review

**DOI:** 10.1007/s10286-020-00683-4

**Published:** 2020-03-26

**Authors:** Phoebe D. Garcia, Jeffrey A. Gornbein, Holly R. Middlekauff

**Affiliations:** 1 David Geffen School of Medicine at UCLA, Los Angeles, CA USA; 2 Department of Medicine, David Geffen School of Medicine at UCLA, Los Angeles, CA USA; 3 Department of Computational Medicine, David Geffen School of Medicine at UCLA, Los Angeles, CA USA; 4 Division of Cardiology, Department of Medicine, David Geffen School of Medicine at UCLA, A2-237 CHS, 650 Charles Young Drive South, Los Angeles, CA 90025 USA

**Keywords:** Autonomic nervous system, Electronic cigarettes, Nicotine, Smoking, Sympathetic nervous system, Vaping

## Abstract

**Purpose:**

Electronic cigarettes (ECs) are the fastest growing tobacco product in the USA, and ECs, like tobacco cigarettes (TCs), have effects on the cardiovascular autonomic nervous system, with clinical implications. The purpose of this review was to collect and synthesize available studies that have investigated the autonomic cardiovascular effects of EC use in humans. Special attention is paid to the acute and chronic effects of ECs, the relative contributions of the nicotine versus non-nicotine constituents in EC emissions and the relative effects of ECs compared to TCs.

**Methods:**

Using the methodology described in the Preferred Reporting Items for Systematic Reviews and Meta-Analysis (PRISMA) statement, we conducted a literature search of the Ovid PubMed and Embase databases on 6 December 2019 using keywords in titles and abstracts of published literature. Acute (minutes to hours) and chronic (days or longer) changes in heart rate variability (HRV), heart rate (HR) and blood pressure (BP) were used as estimates of cardiovascular autonomic effects.

**Results:**

Nineteen studies were included in this systematic review, all of which used earlier generation EC devices. Acute EC vaping increased HR and BP less than acute TC smoking. Nicotine but not non-nicotine constituents in EC aerosol were responsible for the sympathoexcitatory effects. The results of chronic EC vaping studies were consistent with a chronic sympathoexcitatory effect as estimated by HRV, but this did not translate into chronic increases in HR or BP.

**Conclusions:**

Electronic cigarettes are sympathoexcitatory. Cardiac sympathoexcitatory effects are less when vaping using the earlier generation ECs than when smoking TCs. Additional studies of the latest pod-like EC devices, which deliver nicotine similarly to a TC, are necessary.

**Electronic supplementary material:**

The online version of this article (10.1007/s10286-020-00683-4) contains supplementary material, which is available to authorized users.

## Introduction

Electronic cigarettes (ECs), introduced in the USA in 2007, are currently used by over 10 million adults and almost 5 million youths, making them the fastest growing tobacco product available today [10, 50]. Despite the ready availability and widespread use of ECs, little is known about their short- and long-term effects on cardiovascular health [30]. In contrast, it is well known that tobacco cigarette (TC) smoking is the single most important modifiable risk factor for cardiovascular disease in the USA. Approximately 480,000 people in the USA die from TC-related diseases each year, of which cardiovascular disease is the most prevalent cause. As part of a harm reduction strategy, TC smokers have been encouraged to switch to ECs, although it remains unproven and unknown whether the adverse cardiovascular effects of ECs are less than those of TCs. Further, the short- and especially long-term health effects of the epidemic of EC vaping among our youth [17], most of whom have never smoked TCs, is of concern and remains unknown.

TC smoking promotes cardiovascular disease through increases in oxidative stress and inflammation, leading to endothelial dysfunction and platelet activation [1]. TC smoking also has adverse effects on the autonomic nervous system. Specifically, several robust investigative techniques, including microneurography to record direct post-ganglionic sympathetic nerve activity, norepinephrine spillover to determine systemic and/or organ-specific sympathetic activation and heart rate variability (HRV) to determine cardiac sympathetic to parasympathetic balance, have been used to show that TC smoking increases sympathetic nerve activity in humans [13, 33, 37]. Sympathetic activation increases heart rate (HR) and blood pressure (BP), potentially triggering ischemia and arrhythmias [4, 33], and may also promote atherosclerosis [29].

Although most toxicants from smoking are present at orders of magnitude lower—if present at all—in EC vapers than in TC smokers, one toxicant is not, namely, nicotine [19, 20, 31]. Plasma nicotine levels in EC vapers are similar to those in TC smokers [42, 49], and nicotine is a sympathomimetic agent. Several recent studies have reported that ECs, similar to TCs, increase sympathetic nerve activity, as estimated by acute and chronic changes in HR, BP and HRV [2, 5, 8, 15, 16, 26, 34, 35, 44, 47, 52].

It is clear that the autonomic effects of ECs are of clinical importance. The publication of these recent reports, coupled with the widespread and increasing use of ECs, mandate a review and synthesis of the available data relevant to the autonomic cardiovascular effects of acute and chronic EC use in humans. Consequently, we have systematically reviewed the autonomic effects of ECs in humans, as estimated by HR, BP and HRV, based on the methodology described in the Preferred Reporting Items for Systematic Reviews and Meta-Analysis (PRISMA) statement [36]. Special attention was paid to the acute (minutes to hours) and chronic (days or longer) effects of ECs, the relative contributions of the nicotine versus non-nicotine constituents in emissions from ECs and the relative effects of ECs compared to TCs.

## Methods

The PRISMA guidelines were followed during the creation of this systematic review in order to ensure transparency and completeness of the review process.

### Search criteria

A literature search of the Ovid PubMed and Embase databases was conducted on 6 December 2019 for titles and abstracts of published literature containing specific keywords. A librarian from the UCLA Biomedical Library was consulted for recommendations regarding the appropriate search keywords and search strategies. The specific search keywords ultimately decided upon were: “autonomic nervous system” OR “blood pressure” OR “heart rate” OR “sympathetic nerve” OR “sympathetic nervous” OR “vagal” OR “vagus” OR “sympathetic” OR “parasympathetic” AND “electronic nicotine delivery systems” OR “electronic cigarettes” OR “e-cigarettes” OR “vape” OR “vaping.” References from articles and related reviews were perused for additional articles. An additional search of the Ovid Pubmed database using the keywords “microneurography AND electronic cigarettes” and “norepinephrine spillover AND electronic cigarettes” was conducted and yielded no articles.

### Inclusion, exclusion, and study eligibility criteria

Inclusion criteria included publications written in or translated into the English language published in any journal. Given the recent invention and introduction of ECs into global markets, the search for publications included in this systematic review was not limited to a specific time frame since all EC studies have been conducted within the last 10 years. All study designs were included in the search, and no limitations were based on country of publication. Exclusion criteria for published literature included absence of autonomic outcomes, such as HR, BP and HRV. Research studies that only studied cannabidiol (CBD) or tetrahydrocannabinol (THC), both active ingredients in cannabis and/or cannabis liquid in ECs, were also excluded. Experimental studies which only involved animals were excluded since the focus of this review was the effects of ECs on the autonomic nervous system in humans.

### Data extraction and organization

Excel (Microsoft Corp., Redwood, WA, USA) was used for further qualitative synthesis of included articles and for the extraction and organization of data. Excel tables were generated, from which data were extracted on the following: participants, interventions, outcomes, and limitations. The approach to the analysis was organized into two comparisons: (1) TC versus ECs and (2) ECs with nicotine (ECN) versus ECs without nicotine (EC0), and each of these comparisons was further divided by acute (minutes to hours) versus chronic (days or longer) effects. Data from studies were included in more than one analysis if relevant to both; for example, a study involving three exposures, such as ECN, EC0 and TC would be included in the TC versus EC analysis and in the ECN versus EC0 analysis.

### Conflict of interest and sources of bias

Analysis of potential sources of bias included ties to, or funding from, the TC or EC industry. The potential for reporting bias was addressed by using the PRISMA guidelines to write a protocol before beginning the review process and not deviating from this protocol throughout all steps of the systematic review.

### Statistical analysis

Within each comparison (TC vs. EC and ECN vs. EC0), using the extracted data, the summary mean difference and its confidence bounds for a given outcome were computed and combined using the random effects model of Viechtbauer under the R software system [46]. This model assumes heterogeneity. The forest plot entry for each study is the mean difference with the corresponding lower and upper 95% confidence bounds.

## Results

### Study selection

**Fig. 1:**
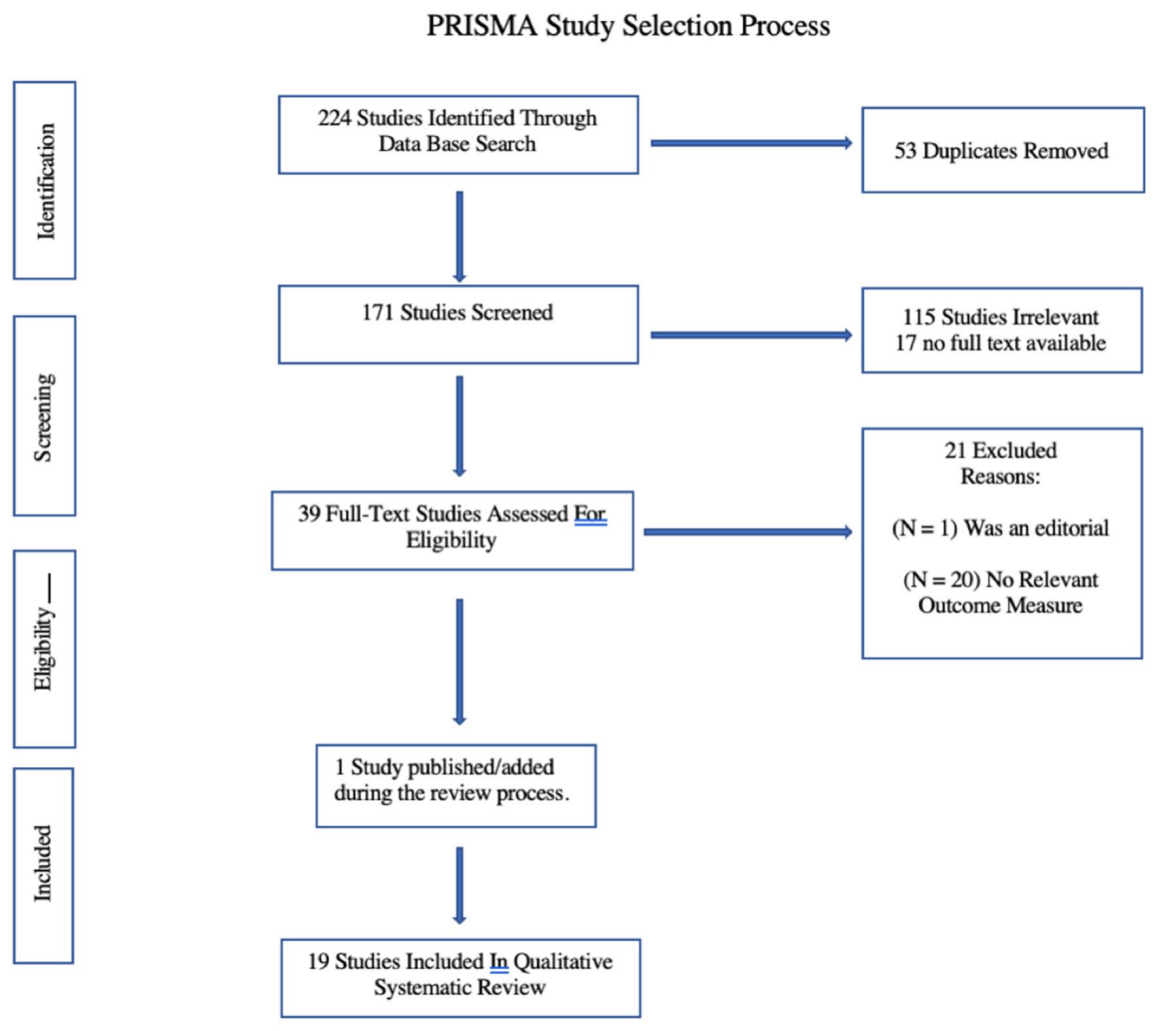
PRISMA (Preferred Reporting Items for Systematic Reviews and Meta-Analysis) study selection process. The electronic search yielded 224 studies, of which 53 were removed because they were found to be duplicates, and 132 studies were excluded because they either did not meet the inclusion criteria (*n* = 115) or a full text was not available (*n* = 17). A full-text review of the remaining 39 studies then led to the exclusion of 21 studies because one study was an editorial and 20 studies had no relevant outcome measures. One additional study [18], published after the literature search, was included while the paper was under review. A total of 19 studies were included in this qualitative systematic review

Two researchers (PDG, HRM) conducted the electronic search for and initial screening of articles, identifying 224 studies for potential inclusion in the systematic review. A further screening of titles and abstracts resulted in the removal of 53 studies because they were found to be duplicates and the exclusion of 132 studies because they either did not meet inclusion criteria (*n* =115) or a full text was not available for review (*n* = 17). A full-text review of the remaining 39 studies led to the exclusion of 21 studies because one study was an editorial and 20 studies had no relevant outcome measures. One additional study [18], published after the literature search, was included while the paper was under review. In total, 19 studies were included in this qualitative systematic review (Fig. 1).

### Acute autonomic cardiovascular effects of TC smoking versus EC vaping

**Fig. 2:**
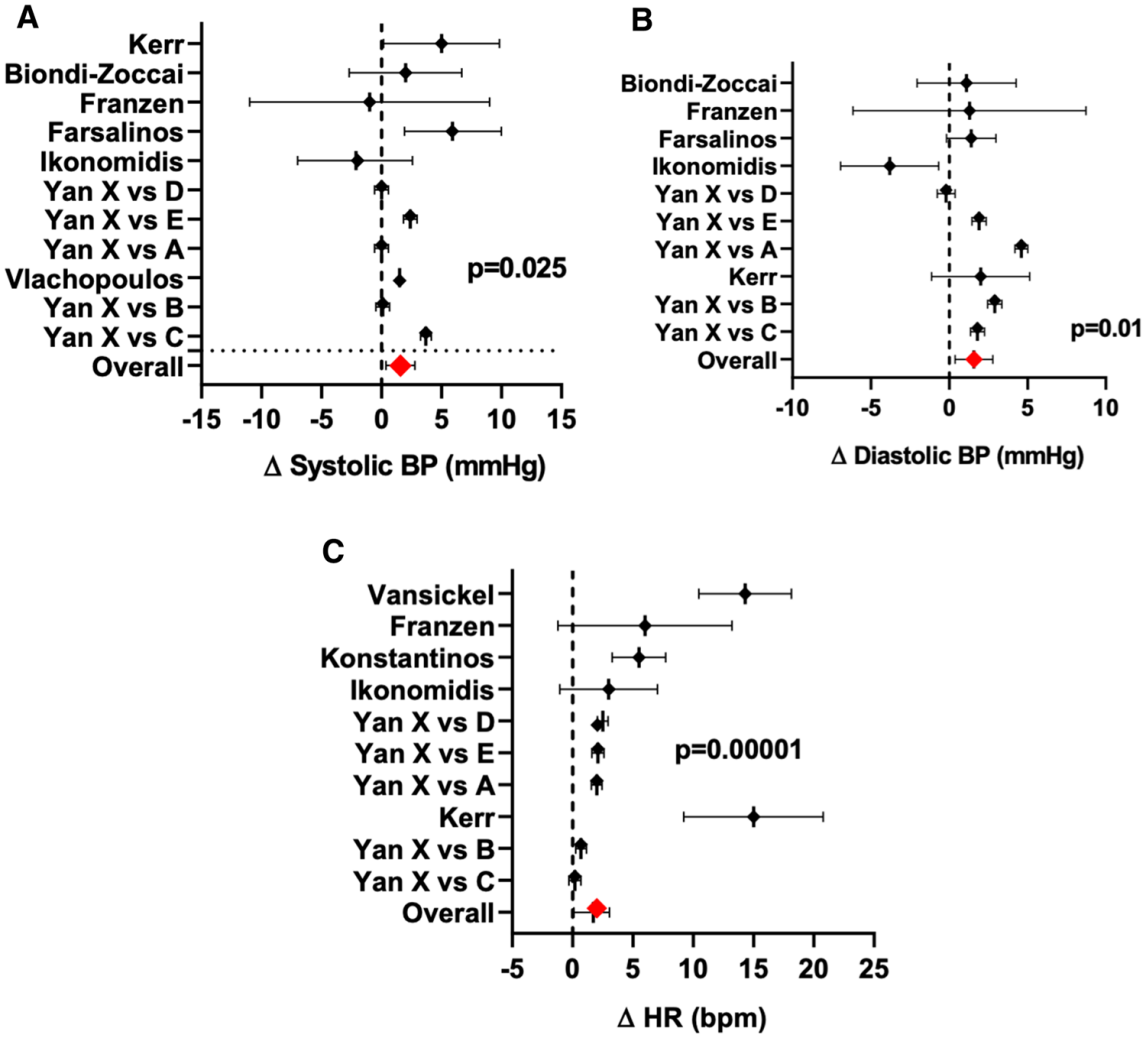
Summary of acute hemodynamic effects of smoking tobacco cigarettes (TCs) versus electronic cigarette (EC) vaping. Data are from 11 comparisons of the acute effects of ECs and TCs on hemodynamic parameters and show that the effects of ECs with nicotine on systolic blood pressure (*SBP*; **a**), diastolic BP (*DBP*; **b**) and heart rate (*HR*; **c**) were significantly less than those of TCs. The forest plot of each study represents the mean differences between TCs and ECs, with the corresponding lower and upper 95% confidence bounds. Plots to the right of the vertical dashed line, which represents no effect, indicate that the effect of TC smoking is worse than than that of EC vaping. The letters A–E in the Yan studies represent different EC devices, and the X represents the TC exposure

Eight studies included in this systematic review investigated the acute effects of TCs versus ECs with nicotine and/or ECs without nicotine on autonomic cardiovascular activity, as estimated by HR, systolic BP (SBP) and diastolic BP (DBP); the results are summarized in Fig. 2 and Electronic Supplementary Material (ESM) Table 1. The difference in the acute effect of TCs compared to the ECs on each variable (SBP, DBP, and HR) in each study is shown in Fig. 2, where the vertical dashed line represents no effect and plots to the right of the vertical dashed line indicate that the TC effect is greater than the EC effect. The results of most studies [5, 15, 26, 44, 52], but not of all studies [16, 25, 47], are consistent with the concept that the effects of TC smoking on acute cardiovascular autonomic functions are greater than those of EC vaping. The overall mean differences between TC smoking and EC vaping across all studies was 1.58 mmHg (95% confidence interval [CI] 0.20–2.97; *p* = 0.025) for SBP, 1.57 mmHg (95% CI 0.37–2.78; *p* = 0.01) for DBP and 3.06 bpm (95% CI 2.01–4.10; *p* = 0.00001) for HR. Unfortunately, most studies did not confirm comparable TC and EC exposures, as estimated by acute increases in plasma nicotine levels. None of the studies included the latest generation pod-like EC device (e.g. Juul). Two studies reported potential biases (authors received or were planning to receive funding from the EC industry) [15, 52]. When these latter two studies were removed from the analysis, the results of the analysis remained essentially unchanged: overall mean differences between TC smoking and EC vaping across all studies was 1.64 mmHg (95% CI 0.09–3.20; *p* = 0.033) for SBP, 2.09 mmHg (95% CI 0.74–3.44; *p* = 0.002) for DBP and 1.86 bpm (95% CI 0.98–2.74; *p* = 0.00001) for HR.

The study of Ikonomidis et al. [25] was the only study that had two phases: an acute phase, which we discuss in this section, and a chronic phase, which we discuss in section Chronic autonomic cardiovascular effects of switching from chronic TC smoking to EC vaping. In the crossover study on acute effects [25], 35 chronic TC smokers were asked to first vape an EC with nicotine (eGo e-cigarette, 1.2% nicotine) for 7 min; then, after a 60-min washout period, they smoked a TC. In the same study, a different cohort of 35 chronic TC smokers first vaped an EC without nicotine for 7 min; then, after a 60-min washout period, they smoked a TC. The results showed that HR and brachial SBP and DBP were unchanged after acute use of the TC or EC with and without nicotine compared to baseline [25]. Plasma nicotine levels following smoking the TC or vaping the EC were not measured.

Biondi-Zoccai et al. [5] studied ECs versus TCs in a randomized crossover trial in 20 chronic TC smokers. These investigators also studied the heat-not-burn-cigarette, but this intervention is beyond the scope and thus not included in this qualitative systematic review. The results of this study show that the smokers who had acutely smoked one commercially available TC had significantly greater increases in SBP and DBP than EC users who vaped nine puffs of an EC (Blu Pro, tobacco flavored, nicotine 16 mg; Imperial Brands, Bristol, UK) [5]. Increases in plasma cotinine levels, but not nicotine levels, were compared after smoking/vaping and were found to increase similarly after acute TC smoking and EC vaping.

In a controlled trial conducted by Farsalinos et al. [15], 36 heavy TC smokers smoked a commercially available TC and 40 chronic EC users vaped an EC with nicotine (11 mg/ml), following which the cardiovascular effects of smoking were compared. SBP and HR, but not DBP, were significantly greater after TC smokers smoked a TC compared to after EC users vaped the EC with nicotine. The increase in plasma nicotine levels was not compared after smoking/vaping [15]. The investigators reported a potential source of bias; they declared that after this study was completed, they received funding from the EC industry. When this study was excluded from the analysis, the results were not significantly changed (see preceding text).

Vansickel et al. [44] measured the HR in a randomized crossover trial in which 32 TC smokers underwent four different exposures in random order: (1) own brand TC; (2) EC with 18-mg nicotine cartridge; (3) EC with 16-mg nicotine cartridge; or (4) sham control (unlit cigarette). HR increased only after acute TC smoking, and not after EC vaping (16- or 18-mg nicotine cartridge) or in the sham-control group. Plasma nicotine only increased after TC smoking, not after EC vaping [44].

Franzen et al. [16] performed a randomized crossover trial in which 15 chronic TC smokers smoked a TC, an EC with nicotine (24 mg/ml) and an EC without nicotine, in random order. SBP, DBP and HR increased similarly after the TC and the EC with nicotine, but not after the EC without nicotine. Changes in plasma nicotine/cotinine were not measured.

Vlachopoulos et al. [47] measured SBP, DBP and HR after 24 TC smokers used either a TC or an EC with an unspecified amount of nicotine for 5 min and 30 min in a randomized crossover trial. HR, SBP, and DBP increased similarly after smoking the TC and vaping the EC for 30 min, but not 5 min [47]. Acute changes in plasma nicotine levels were not measured.

Yan et al. [52] conducted a randomized crossover trial with 23 chronic TC smokers who were clinically confined for the 11-day study and used five different ECs (2 commercially available ECs and 3 non-commercially available ECs—A, B, C, D, E in Fig. 2) and one commercially available TC, in random order, with each session separated by a 36-h washout period. Increases in HR, SBP and DBP were significantly greater after smoking the TC than after vaping the EC [52]. Plasma nicotine after TC smoking had a steeper slope of increase and remained higher than did the plasma nicotine level after acute EC vaping [52]. The investigators reported a potential source of bias; they declared that they received funding from the EC industry. When this study was excluded from the analysis, the results were not significantly changed (see preceding text).

Kerr et al. [26] performed a randomized crossover study in 20 chronic TC who smoked a TC and vaped an EC with 18 mg/ml of nicotine in random order. The increase in HR and SBP, but not DBP, was significantly greater after smoking the TC compared to vaping the EC [26]. Plasma nicotine levels were not measured.

### Acute autonomic cardiovascular effects of ECN versus EC0 vaping

**Fig. 3:**
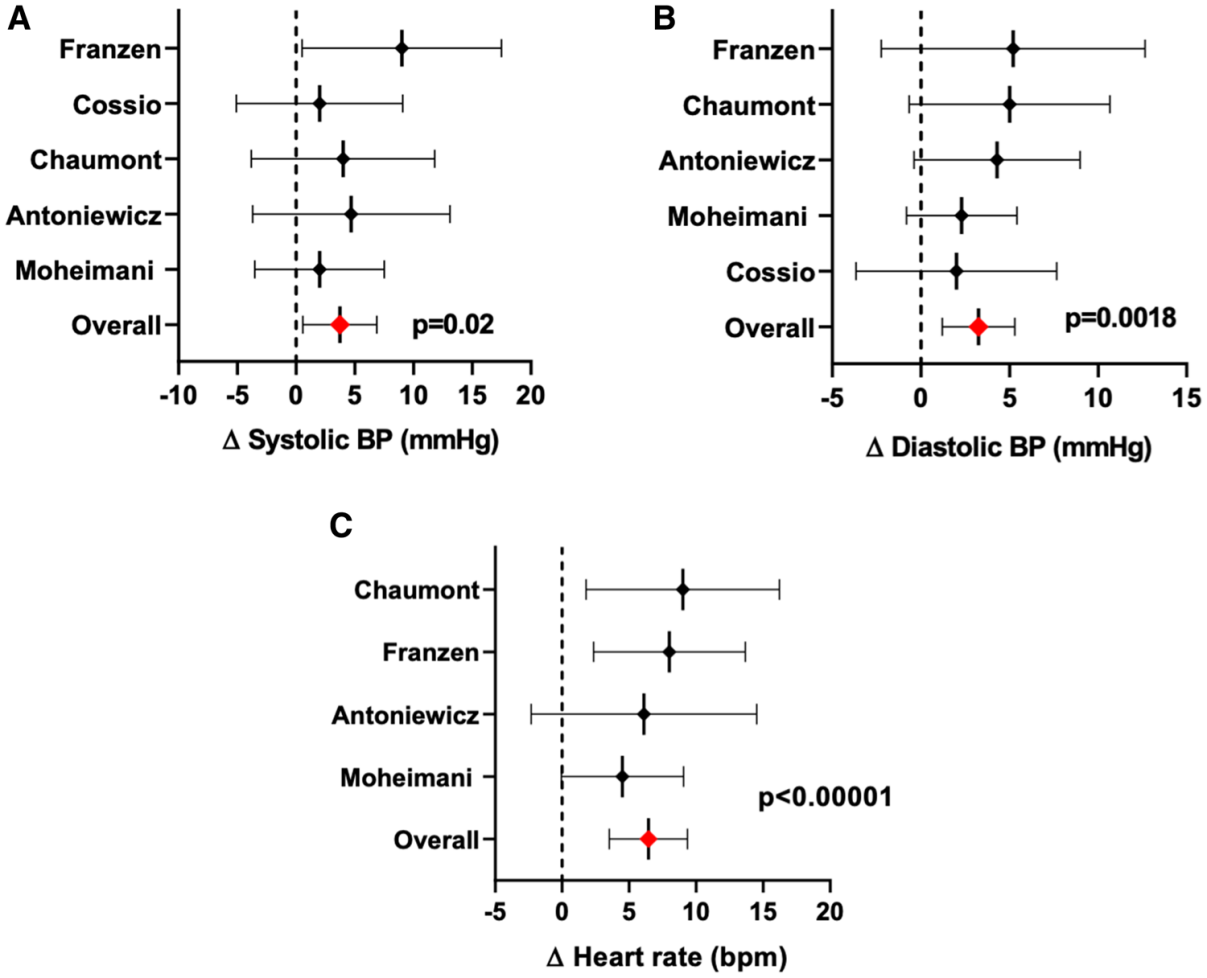
Summary of acute hemodynamic effects of electronic cigarettes with nicotine compared to electronic cigarettes without nicotine. Data from 5 acute studies revealed that the effects of EC with nicotine compared to ECs without nicotine on SBP (**a**), DBP (**b**) and HR (**c**) were significantly less. The forest plot entry for each study is the mean differences between ECN and EC0, with the corresponding lower and upper 95% confidence bounds. The plots to the right of the vertical line of no effect indicate that ECN is worse than EC0. *DBP* diastolic blood pressure, *ECN* electronic cigarette with nicotine, *EC0* electronic cigarette without nicotine, *HR* heart rate, *SBP* systolic blood pressure, *TC* tobacco cigarette

Five studies were included in this systematic review that compared the effects of ECs with nicotine and/or ECs without nicotine or the solvents alone on the autonomic nervous system, as summarized in Fig. 3 and ESM Table 2. The difference in the acute effects of the ECN compared to the EC0 on each variable (SBP, DBP, and HR) in each study is shown in Fig. 3. The vertical dashed line represents no effect, and plots to the right of the vertical dashed line indicate that the effect of ECN is greater than that of EC0. The results of most [8, 16, 34] but not all [2, 9] of these acute vaping studies were consistent with the notion that nicotine—and not the non-nicotine constituents—in the EC aerosol was responsible for the acute sympathomimetic effects of EC vaping. The overall mean differences between the ECN and EC0 studies across all studies was 3.73 mmHg (95% CI 0.59–6.87; *p* = 0.02) for for SBP, 3.25 mmHg (95% CI 1.21–5.30; *p* = 0.0018) for DBP and 6.44 bpm (95% CI 3.52–9.36; *p* < 0.00001) for HR. No author involved in these studies declared any potential financial biases (ESM Table 2; Fig. 3).

Only one study, that conducted by Moheimani et al. [34], measured HRV as an estimate of cardiac sympathetic activity. In this study, the effects of acute EC vaping with nicotine (1.2%) and EC vaping without nicotine (0%) on cardiac sympathetic to parasympathetic balance in 33 healthy nicotine-naive participants was measured. In this open label, randomized, crossover study, each participant vaped an EC with nicotine, an EC without nicotine and a sham control (empty EC) in random order. Only the EC with nicotine, but not the EC without nicotine or sham control, caused an acute sympathomimetic effect as estimated by HRV. HR, but not SBP or DBP also increased significantly only after vaping the EC with nicotine. Plasma nicotine levels were measured immediately after EC use in this study, and the increase in plasma nicotine levels was directly related to the increase in cardiac sympathetic activity, as well as to the increases in SBP and HR [34].

Chaumont et al. [8] performed a randomized, single-blind crossover study in 25 young healthy TC smokers in which TC smokers vaped an EC with nicotine (3 mg/ml), an EC without nicotine, and a sham-control in random order. SBP, DBP, and HR increased significantly only after using the EC with nicotine, not after the EC without nicotine, or the sham-control [8]. Plasma nicotine levels increased after vaping the EC with nicotine, but the increase did not correlate with changes in HR or BP.

Franzen et al. [16] conducted a randomized crossover trial in 15 chronic TC smokers before and after vaping an EC with nicotine (24 mg/ml) and an EC without nicotine and found that peripheral SBP was significantly increased after vaping the EC with, but not without, nicotine [16]. Plasma nicotine levels were not measured.

In contrast to these 3 trials, Cossio et al. [9] conducted a randomized single-blinded trial of 16 young healthy nicotine-naïve participants, and found no significant changes in HR, SBP, DBP after vaping an EC with nicotine (18 puffs in 6 min) or without nicotine [9]. An increase in plasma nicotine levels was not measured, or confirmed, in this trial in nicotine-naïve participants [9].

Surprisingly, and uniquely, a double-blinded cross-over study conducted in 17 healthy occasional TC smokers reported that SBP, DBP and HR increased after vaping an EC with nicotine (19 mg/ml), and SBP and DBP, but not HR, increased similarly after vaping an EC without nicotine [2]. This is the only trial comparing ECs with and without nicotine in which the EC without nicotine significantly increased BP, although it remains uncertain that this pressor effect was mediated by an increase in sympathetic vasoconstriction.

### Chronic autonomic cardiovascular effects of ECs

Robust techniques by which to measure chronic autonomic cardiovascular system activity include HRV, direct sympathetic nerve activity directed to the muscle vasculature (e.g. using microneurography [37]) and norepinephrine spillover, either systemic or directed to specific organs and tissues [13]. To date, no studies have measured muscle sympathetic nerve activity or norepinephrine spillover results in acute or chronic EC use. The use of HR and BP as surrogate endpoints for changes in autonomic cardiovascular effects is less secure in the chronic compared to the acute setting due to many potential confounders.

**Fig. 4:**
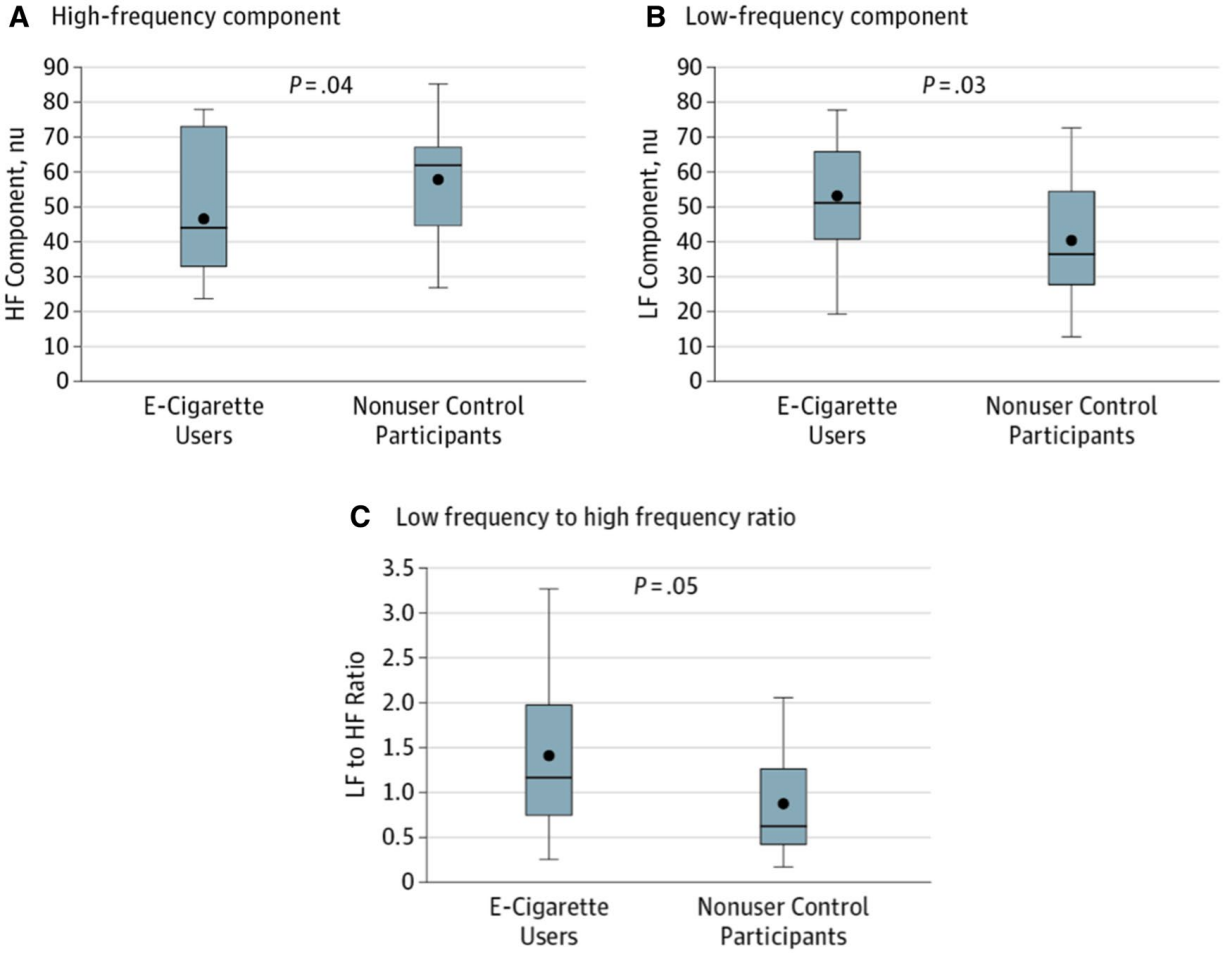
Boxplot of heart rate variability in chronic EC vapers compared to age-matched controls.** a **The high-frequency (*HF*) component, an indicator of vagal activity, was significantly decreased in the EC vapers compared with non-user control individuals (mean [SEM] 46.5 [3.7] vs. 57.8 [3.6] nu; *p* = 0.04). **b**, **c** The low-frequency (*LF*) component, an indicator largely of sympathetic activity (mean [SEM] 52.7 [4.0] vs. 39.9 [3.8] nu, *p* = 0.03), and the LF to HF ratio (1.37 [0.19] vs. 0.85 [0.18]; *p* = 0.05), were significantly increased in the EC users compared with the non-user controls. These results are consistent with sympathetic predominance. These findings were present even in the absence of recent EC use, as verified by the absence of detectable nicotine in the plasma. *SEM* Standard error of the mean. Filled circles represent the mean, horizontal lines represent the median. Used with permission from Moheimani et al. [35], copyright 2017, American Medical Association

Our literature search uncovered two studies [35, 38] conducted in chronic EC users who did not also smoke TCs (no dual users); HRV [35] and HR and BP [38] were the primary autonomic outcomes measured [35, 38]. In the first study, which enrolled 23 chronic EC vapers and 19 non-vaper controls [35], HRV was used to determine the cardiac sympathetic to parasympathetic balance in EC vapers who refrained from vaping for 12 h immediately prior to the study, which was confirmed by non-detectable plasma nicotine levels at the time of the study. Chronic EC users exhibited sympathetic predominance compared to similarly-aged non-vaper controls (Fig. 4). There was no difference in resting HR or BP. In a second, 3.5-year prospective study [38], HR and BP were periodically measured in nine chronic EC vapers who refrained from vaping for 60 min immediately before HR and BP measurements were made. Twelve nicotine-naïve participants were enrolled as age- and sex-matched controls. No differences in HR or BP were found in EC vapers compared to baseline or compared to nicotine-naïve controls, over time. In summary, these data support the concept that EC smokers have chronically elevated cardiac sympathetic activation compared to non-vapers, as measured by HRV, but that this cardiac sympathetic activation does not translate into clinically detectable higher HR or BP.

### Chronic autonomic cardiovascular effects of switching from chronic TC smoking to EC vaping

Our search uncovered six TC to EC switch studies in chronic TC smokers. These studies reported HR and BP outcomes, summarized in the following paragraph and in ESM Table 3. Only one of these studies was a short-term (5 day) switch study [11] during which the participants were confined and smoking behavior was monitored. In total, 105 chronic TC smokers were randomized to three general groups: (1) TC to EC complete switch; (2) TC to EC switch but TCs were allowed; (3) complete TC and nicotine cessation. The EC flavors included cherry and tobacco. At 5 days after study initiation, there were no significant overall changes in HR or BP reported (ESM Table 3).

The remaining five studies were long-term switch studies. Farsalinos et al. [14] followed 300 TC smokers who were enrolled in a prospective 1-year randomized switch study that compared ECs with 2.4% nicotine, ECs with low levels (1.8%) of nicotine and ECs with no (0%) nicotine. Most participants continued to use TCs during the study. Of the 183 participants who completed the study, a slight but significant decrease in SBP and DBP, but not HR, was reported. Unfortunately, there was no time control, so whether this reduction in BP was attributable to the switch from TCs to ECs or due to the participants becoming more comfortable with the study procedures is not known. In a retrospective analysis of medical records of 43 hypertensive TC smokers who reduced their TC smoking and/or switched to ECs with the aim to quit smoking, Polosa et al. [39] found that there was a significant reduction in SBP and DBP. There was no change in BP in the chronic TC control group. Ikonomidis et al. [25] enrolled 70 TC smokers in an EC switch study, confirming TC cessation by periodic monitoring of exhaled carbon monoxide (CO). Twenty age-matched TC smokers served as a time control. At 1 year, SBP, but not DBP or HR, was significantly lower in the compliant and partially compliant participants compared to baseline values. BP and HR did not change in the TC smoking controls. George et al. [18] conducted a randomized controlled trial of TC smokers randomized to ECs with nicotine or ECs without nicotine for 1 month. Approximately 50% of those who switched to ECs, either with or without nicotine, continued to smoke TCs, as estimated by elevated exhaled CO levels at follow-up. Another cohort of TC smokers who did not want to quit were followed in a parallel preference cohort. When adjusted for baseline variables, there was no difference in BP or HR in either of the EC arms compared to the TC arm. Finally, Veldheer et al. [45] enrolled 263 chronic TC smokers into a switch study involving the switch to ECs or a non-EC substitute. Although the majority of participants had reduced the number of TCs smoked each day, almost all were still smoking TCs at 3 months. At 3 months, similar to the the results of the George et al. [18] switch study, there was no decrease in HR or BP. In summary, most [14, 25, 39], but not all [18, 45] of the TC to EC switch studies involving chronic TC smokers tended to show a small decrease in BP, but not HR, over time.

## Discussion

In this systematic review of 19 studies which used the earlier generation EC devices, the findings support the notion that acute EC vaping has acute effects on the autonomic cardiovascular system; more specifically, there are increases in sympathetic excitation as estimated by acute increases in HRV, HR and BP. Further, the sympathoexcitatory effects of acute EC use are largely attributable to nicotine—and not to the non-nicotine constituents in EC emissions [2, 8, 16, 34]. Additionally, these sympathoexcitatory effects of acute EC vaping are less than those of acute TC smoking, as estimated by HR and BP [5, 15, 16, 25, 26, 44, 47, 52]. Finally, limited data suggest that chronic EC use is associated with sympathetic activation [35], even in the absence of acute nicotine inhalation, although more data are needed for confirmation.

### Acute EC vaping increases sympathetic nerve activity: a nicotine effect

Several studies of acute vaping included in this review compared the autonomic cardiovascular effects of ECs with and without nicotine [2, 8, 9, 16, 34]. All but two of these studies showed that acute increases in HRV, HR and/or BP, surrogates for sympathetic nerve activity, were significantly greater after using the ECs with nicotine compared to ECs without nicotine. Increases in plasma nicotine levels after EC vaping with nicotine were not confirmed in either of the two negative studies [2, 9]. In fact, in one of these two studies, the participants were non-smokers and had never used an EC prior to the study [9]. Ineffective nicotine delivery has been reported in inexperienced EC vapers using ECs for the first time—especially when early-generation devices were used [7]. It is conceivable that the participants in this study were not exposed to significant EC aerosol. In another study, the acute effects of vaping the solvents propylene glycol and vegetable glycerol alone, without nicotine or flavorings, were studied [6]. The results showed that vaping the solvents alone did not increase the HR and BP, consistent with previous data that it is the nicotine, and not the non-nicotine constituents, in the EC aerosol that acutely increase sympathetic nerve activity.

### Mechanisms of acute sympathetic excitation with ECNs

The mechanisms underlying the acute sympathomimetic effects of inhaled nicotine are complex. Nicotine has direct pharmacological effects on peripheral post-ganglionic nerve endings, resulting in increased exocytotic norepinephrine release [22]. Norepinephrine release in cardiac tissue interacts with β-adrenergic receptors to increase the HR and contractility; exocytotic norepinephrine release in vascular tissue binds to α-adrenergic receptors, causing vasoconstriction [22].

Nicotine may also acutely increase post-ganglionic sympathetic nerve firing, which can be directly recorded using the technique of peroneal microneurography [37]. However, this peripheral sympathetic nerve excitation may be suppressed in young people, in whom the pressor effect, mediated by exocytotic norepinephrine release, activates inhibitory baroreflexes in a negative feedback loop. The baroreflexes exert an acute sympathoinhibitory effect, thereby restoring BP towards its normal level [33]. Studies in TC smokers have confirmed that baroreflex activation by the pressor responses masks the increase in post-ganglionic sympathetic nerve traffic following acute TC smoking [21, 37]. Pharmacological strategies to prevent the pressor response have been utilized to unmask increased post-ganglionic sympathetic nerve activity recorded with microneurography following TC smoking [37]. In older smokers in whom the baroreflexes may be attenuated, TC smoking is accompanied by increased sympathetic traffic [23, 24]. Thus, although the sympathoexcitatory effects of smoking may be reflexively suppressed in healthy young people in whom baroreflexes are intact, sympathoexcitation may be exaggerated—and thus potentially more lethal—in older smokers in whom baroreflex function is attenuated.

### Cardiovascular sequelae of acute increases in sympathetic nerve activity during smoking

The clinical importance of acute increases in sympathetic nerve activity associated with EC vaping may be extrapolated from TC smoking studies. Periodic, recurrent increases in sympathetic nerve activity may be accompanied by abrupt increases in HR and BP, which occur throughout the day. Recurrent bouts of hypertension may contribute to endothelial injury [3]. Additionally, increases in HR and BP increase myocardial oxygen demand [4]. This augmented demand may further be exacerbated by decreased nutrient supply since TC smoking also causes sympathetically-mediated coronary vasoconstriction and vasospasm [51]. Finally, acute sympathetic activation may trigger atrial and ventricular arrhythmias in TC smokers [27, 41].

### Acute sympathoexcitatory effects are less following acute EC vaping compared to acute TC smoking

Several studies included in this review compared the acute autonomic cardiovascular effects of TCs and ECs and found that the acute effects of ECs, including acute increases in HR and BP, were significantly less than the acute effects of TC smoking. However, there are several limitations to these studies that must be acknowledged. First, HR and BP, outcomes indicative of sympathetic excitation, were not the pre-specified primary outcomes in most of these studies (ESM Table 1). Second, although the studies sought to compare TCs to ECs, it remains uncertain if the exposures to these tobacco products were, in fact, comparable, as estimated by increases in plasma nicotine. Plasma nicotine levels were not measured in many of the studies to confirm similar exposures. Additionally, investigations of ECs used a wide variety of EC devices, reflecting the rapidly evolving innovations in EC technology. Whereas early studies used devices that deliver nicotine with inferior pharmacokinetics compared to TCs, studies using the pod devices would be expected to deliver greater quantities of nicotine at a faster rate, resembling the pharmacokinetics of nicotine delivery by a TC. Additional studies comparing TC smoking to the pod EC (Juul) with measurement of pre/post-nicotine levels are therefore necessary.

The protocols in these laboratory investigations, by necessity, utilized a relatively short, intense vaping period, such as 60 puffs of an EC in 30 min. However, these protocols may not replicate how individuals consume their tobacco product outside of the laboratory—especially their ECs. A TC burns and, therefore, TC smokers typically smoke one TC in several rapid puffs over 7–10 min. In contrast, ECs do not burn and therefore may be used in a much less concentrated manner—with single, individual puffs taken randomly throughout the day, interspersed by large intervals. In fact, there is no standard or “normal” vaping topography. While it would appear that people use their tobacco device to achieve and maintain a certain satisfying level of nicotine [42], the profile of vaping behavior to achieve that nicotine level may be quite variable.

Finally, the participants in these studies varied from nicotine-naïve/never smokers to chronic EC vapers/chronic TC smokers, and this heterogeneity in experience with ECs could be expected to impact both the efficiency of aerosol delivery during EC use, as well as the physiologic effect of the aerosol on hemodynamics. The length and strength of the inhalation effort required to use an EC is quite different from that required to smoke a TC, and thus those inexperienced with an EC device tend to receive a lower aerosol exposure [7]. Furthermore, chronic TC smoking leads to stiffening of the vasculature [48]; it is conceivable that the effects of comparable vaping sessions may have different hemodynamic effects in a chronic TC smoker, in whom the arteries are less compliant, compared with a non-TC smoker. In summary, there are several limitations inherent in the available data and thus in the strength of its interpretation.

Based on the much lower levels, or even complete absence, of carcinogens and toxicants in EC aerosol compared with TC smoke, ECs have been promoted as a harm reduction strategy in TC smokers. However, it remains uncertain that the cardiovascular effects of EC use, especially use of the newer pod-like devices which deliver nicotine with similar pharmacokinetics as TCs, will lead to significant harm reduction in cardiovascular disease.

### Chronic sympathoexcitation in chronic EC vapers

In addition to these acute increases in sympathetic nerve activity, the results of one small study are consistent with the concept that chronic EC vaping is associated with chronically elevated cardiac sympathetic activity, as estimated by HRV; the HR and BP were not different [35]. Unfortunately, in this small study, there was no chronic TC smoking group, so it remains unknown whether cardiac sympathetic nerve activation was similar in chronic EC users and chronic TC smokers. No studies were identified that utilized the powerful and specific techniques of microneurography or norepinephrine spillover to assess the chronic autonomic effects of ECs [13, 37].

### Mechanisms of chronic sympathoexcitation in smokers

Tobacco cigarette smoking is associated with a hyperadrenergic state, and potential mechanisms have been proposed [33]. In addition to the intermittent sympathetic excitation associated with increases in nicotine throughout the day, sympathetic nerve activity may be elevated even in the absence of acute exposure. The amygdala, a brain region in which nicotine receptors are present and which integrates autonomic responses to stress and addiction, has been found to be abnormal in TC smokers [40, 53]. Amygdalar dysregulation may further contribute to the sympathomimetic effects of smoking and, importantly, amygdalar dysregulation has been associated with increased cardiovascular risk [43].

### Cardiovascular sequelae of chronic sympathoexcitation

The chronic hyperadrenergic state in EC users may contribute to the development of inflammatory atherosclerosis as part of an integrated network called the “Splenocardiac Axis.” Evidence supports the concept that the brain (amygdala) [43], autonomic nervous system and hematopoietic tissues (bone marrow and spleen) are linked in the development of atherosclerosis and myocardial infarction. In this model, norepinephrine released from sympathetic nerves binds to β-3 adrenergic receptors on mesenchymal stem cells [32] to mobilize hematopoietic progenitor cells, which in turn migrate from the bone marrow to the spleen [28] where they multiply in response to growth factors. Augmented numbers of pro-inflammatory monocytes enter the circulation and reach the arterial wall where increased monocyte recruitment coupled with pro-oxidative and pro-thrombotic factors promote atherosclerosis [12, 29].

### Limitations

The focus of this review was the autonomic cardiovascular effects of ECs, but our literature search identified almost no studies of direct autonomic cardiovascular effects. Accordingly, we used HR and BP as surrogates. Applying these terms, our search yielded 224 articles. Nonetheless, additional relevant articles may have been missed. Many of the included studies were on acute EC use. Acute EC use, which increases sympathetic nerve activity acutely, thereby potentially triggering arrhythmias and ischemia, may have only limited value in providing information on longer term autonomic effects. Finally, although we have discussed the potential atherogenic effect of chronic EC use, which is attributable to its sympathomimetic and inflammatory effects (splenocardiac axis), the long-term use of smokeless tobacco (e.g. snus or chewing tobacco) has not been shown to cause early atherosclerosis. However, smokeless tobacco may have only limited relevance to the potential for inhaled nicotine to cause atherogenic effects. Due to differing pharmacokinetics and sites of delivery, ECs may have greater sympathoexcitatory effects, thereby potentially promoting pro-inflammatory effects; their effects on early atherosclerosis remain to be determined.

## Conclusions

The articles included in this systematic review support the notion that ECs have acute sympathoexcitatory effects that are attributable to the nicotine in EC aerosol and not to the non-nicotine constituents. Further, these sympathoexcitatory effects appear to be reduced compared to those associated with TC smoking. However, due to the rapidly evolving EC technology and changing pharmacokinetics of nicotine delivery, one must be cautious in concluding that sympathoexcitatory effects of ECs are less than than those of TCs. Additional, rigorous studies comparing indices of autonomic cardiovascular effects of TC smoking with the latest generation pod-like ECs (Juul), accompanied by measurements of plasma nicotine levels, are necessary.

## Electronic supplementary material

Below is the link to the electronic supplementary material.

Supplementary file1 (PDF 233 kb)
