# Supplementary material for: Cardiovascular autonomic effects of electronic cigarette use: a systematic review
Source: Clin Auton Res. 2020 Mar 26;30(6):507–19. doi: 10.1007/s10286-020-00683-4 (PMC7704447; doi:10.1007/s10286-020-00683-4)
Supplement: Supplementary file 1 — Supplementary file1 (PDF 233 kb) [file 10286_2020_683_MOESM1_ESM.pdf]

Table 1 Study Characteristics: Acute Autonomic Effects of TC's Vs EC's

Author & Year	Study Design	Sample Size	Acute Exposure	Exposure Device (Device Type)	Nicotine Concentration	Duration Of Exposure	Nicotine/Cotinine Levels	Autonomic CV Outcomes	CVO Primary Outcomes	Conflict of Interest
Biondi-Zoccai et al, 2019 [5]	Randomized crossover study	N = 20 TC smokers	20 TC smokers<br><br>20 ECN vapers	Marlboro Gold<br>EVC Blu-Pro	Marlboro Gold = .60mg per TC<br><br>ECN Blu-Pro = 16 mg nicotine cartridge equivalent to .58mg in 9 puffs	TC - 1 cigarette<br><br>ECN - 9 puffs	Serum Cotinine levels	SBP<br>DBP	Not Primary Endpoint	No
Farsalinos et al, 2014 [15]	Randomized controlled trial	N = 76 TC smokers and ECN users	36 TC (not-crossover study)<br>40 ECN (not-crossover)	TC - commercially available<br><br>ECN eGo-T battery Nobacco with an eGo-C atomizer, 2nd gen	TC = 1 mg<br><br>ECN= 11 mg/ml nicotine cartridge	TC - 1 cigarette<br><br>ECN - used for 7 mins	Not measured	SBP<br>DBP<br>HR	Not reported	Yes
Franzen et al, 2018 [16]	Randomized double-blind crossover study	N = 15 TC smokers	15 TC<br><br>15 ECN<br><br>15 EC0	TC - Philip Morris cig.<br><br>ECN - DIPSE eGo-T battery<br><br>CE4, 3rd gen<br><br>EC0 - DIPSE eGo-T battery CE4, 3rd gen	TC = not reported<br>ECN = 24 mg/mL nicotine cartridge<br><br>EC0 = 0 mg/ml nicotine cartridge	TC - 1 cigarette<br>ECN - one puff every 30 seconds for 10 (4sec) puffs,<br><br>EC0 - one puff every 30 seconds for 10 (4sec) puffs	Not measured	Central SBP<br>Central DBP<br>Peripheral SBP<br>Peripheral DBP<br>HR	Not reported	No
Ikonomidis et al, 2018 [25]	Randomized crossover study	N = 70 TC smokers	N = 70 TC<br><br>N = 35 ECN<br><br>N = 35 EC0	TC - did not say brand, conventional cigarette<br><br>ECN - eGo Epsilon BDC 1100, Nobacco TM<br><br>EC0 - eGo Epsilon BDC 1100, Nobacco TM	TC = not reported<br>ECN = 12 mg/mL nicotine cartridge<br><br>EC0 = 0 mg/ml nicotine cartridge	TC - 1 cigarette<br>ECN - 7 mins<br>EC0 - 7 mins	Not measured	Central SBP<br>Central DBP<br>Peripheral SBP<br>Peripheral DBP<br>HR	Not reported	No

Kerr et al, 2019 [26]	Randomized non-blinded crossover study	N = 20 TC smokers	N = 20 TC<br><br>N = 20 ECN	TC - commercially available<br><br>ECN - SmokeMax Groove Trading Ltd, 1300mAh variable voltage rechargeable battery, a tank and an atomizer, 2nd gen	TC = approximately 0.5mg of nicotine<br><br>ECN = 17.27 mg/ml nicotine cartridge	TC - 1 cigarette<br>ECN - 15 puffs, approximately equivalent to .5mg	Not measured	SBP<br>DBP<br>HR	Not reported	No
Vlachopoulos et al, 2016 [47]	Crossover clinical study	N = 24 TC smokers	N = 24 TC<br><br>N = 24 ECN<br><br>N = 24 sham	TC - not reported<br><br>ECN - not reported<br><br>sham - not reported	TC = not reported<br><br>ENC = not reported<br><br>Sham = not reported	TC - 5 min<br><br>EC - 5 min<br><br>EC - 30 min<br><br>sham - nothing for 60 min	Not measured	SBP<br>DBP<br>HR	Not reported	No
Vansickel et al, 2010 [44]	Randomized crossover study	N = 32 TC smokers	N = 32 TC<br><br>N = 32 Hydro - ECN<br><br>N = 32 NPRO - ECN<br><br>N = 32 Sham	TC - participants used their own commercially available TC<br><br>ECN – Hydro<br><br>ECN - NPRO<br><br>Sham - unlit TC	TC - not reported<br><br>ECN - Hydro 18 mg cartridge<br><br>ECN - NPRO 16 mg cartridge<br><br>Sham - unlit TC	TC - 1 cigarette<br>ECN - Hydro 10 puffs<br><br>ECN - NPRO 10 puffs<br><br>Sham - unlit TC	Plasma Nicotine	HR	Not reported	No
Yan et al, 2015 [52]	Randomized single-blinded, 6 period crossover trial	N = 23 TC smokers	N = 23 Product A<br><br>N = 23 Product B<br><br>N = 23 Product C<br><br>N = 23 Product D<br><br>N = 23 Product E<br><br>N = 24 Product F	Product A - non-commercial ECN rechargeable cartomizer<br><br>Product B - non-commercial ECN rechargeable cartomizer<br><br>Product C - non-commercial ECN Magnificent Menthol rechargeable cartomizer<br><br>Product D - commercially	Product A - 2.4% nicotine, 75% Gly<br><br>Product B - 2.4% nicotine, 50% Gly, 20% PG<br><br>Product C- 2.4%, 75% Gly<br><br>Product D - 1.6% nicotine, 75% Gly<br><br>Product E - 1.6% nicotine, 50% Gly, 20% PG	Product A - 50 puffs (5 sec puffs at 30 sec intervals)<br><br>Product B - 50 puffs (5 sec puffs at 30 sec intervals)<br><br>Product C - 50 puffs (5 sec puffs at 30 sec intervals)<br><br>Product D - 50 puffs (5 sec puffs at 30 sec intervals)	Plasma Nicotine	SBP<br>DBP<br>HR	Not reported	Yes

				available rechargeable cartomizer Blu E-cig<br><br>Product E - Classic Tobacco ECN rechargeable cartomizer<br><br>Product F - Marlboro Gold King Size	Product F- not reported	Product E - 50 puffs (5 sec puffs at 30 sec intervals)<br><br>Product F - 1 entire TC				

Abbreviations:

CV = cardiovascular
CVO = cardiovascular outcome
DBP=diastolic blood pressure
EC - electronic cigarette
ECN - electronic cigarette with nicotine
EC0 - electronic cigarette without nicotine
Gly - Glycerin
HR - heart rate
PG - Propylene Glycol
SBP = systolic blood pressure
TC - tobacco cigarette

Table 2 Study Characteristics: Acute Autonomic Cardiovascular Effects of ECs with Nicotine vs ECs without Nicotine

Author & Year	Study Design	Sample Size	Acute Exposure	Exposure Device (Device Type)	Nicotine Concentration	Duration Of Exposure	Nicotine/Cotinine Levels	Autonomic CV Outcomes	CVO Primary Outcomes	Conflict of Interest
Franzen et al, 2018 [16]	Randomized double-blind crossover study	N = 15 TC smokers	N = 15 TC<br><br>N = 15 ECN<br><br>N = 15 EC0	TC - Philip Morris<br>ECN - DIPSE eGo-T battery CE4, 3rd gen<br>EC0 - DIPSE eGo-T battery CE4, 3rd gen	TC = not reported<br>ECN = 24 mg/mL nicotine cartridge<br>EC0 = 0 mg/ml nicotine cartridge	TC - 1 cigarette<br>ECN - one puff every 30 seconds for 10 puffs (4 sec inhalations)<br>EC0 - one puff every 30 seconds for 10 puffs, (4 sec inhalations)	Not measured	Central SBP<br>Central DBP<br>Peripheral SBP<br>Peripheral DBP<br>HR	Not reported	No
Antoniewicz et al, 2019 [2]	Randomized double-blind crossover study	N = 17 Occasional TC smokers	N = 17 ECN<br><br>N = 17 EC0	ECN - eVic-VT, Shenzhen Joy-Etech, 3rd gen<br>EC0 - eVic-VT, Shenzhen Joy-Etech, 3rd gen	ECN = 19 mg/ml<br>EC0 = 0 mg/ml	ECN = 30 puffs for 30 mins<br>EC0 = 30 puffs for 30 mins	Baseline plasma cotinine only	SBP<br>DBP<br>HR	Not reported	No
Chaumont et al, 2018 [8]	Randomized single-blind crossover study	N = 25 TC Smokers	N = 25 ECN<br><br>N = 25 EC0<br><br>N = 25 Sham EC	ECN - last-generation high-power EC with commercially available parts in U.S (Smoke©, Shenzen, China)<br><br>EC0 - last-generation high-power EC with commercially available parts in U.S (Smoke©, Shenzen, China)<br><br>Sham EC - last-generation high-power EC with commercially available parts in U.S (Smoke©, Shenzen, China) while turned off	ECN = 3 mg/ml<br><br>EC0 = 0 mg/ml<br><br>Sham EC = turned off	ECN = 25 puffs (4sec inhalations at 30sec intervals)<br><br>EC0 = 25 puffs (4sec inhalations at 30sec intervals)<br><br>Sham = 25 puffs (4sec inhalations at 30sec intervals)	Plasma nicotine	SBP<br>DBP<br>HR	No, secondary outcome	No

Cossio et al, 2019 [9]	Single-blind randomized study	N = 16 Nicotine naïve participants	N = 16 ECN<br><br>N = 16 EC0<br><br>N = 16 Cigarette like pipe	ECN - Combination of a battery Cirrus 3, White Cloud Cigarette and cartridge<br><br>EC0 - Combination of a battery Cirrus 3 and cartridge Cigarette like pipe - Harmless Cigarette Quit Smoking Aid	ECN - 5.4% nicotine<br><br>EC0 - 0% nicotine Harmless Cigarette Quit Smoking Aid - 0% nicotine	ECN - 6 mins, 18 puffs (4 sec inhalations)<br><br>EC0 - 6 mins, 18 puffs (4 sec inhalations) Harmless<br><br>Cigarette Quit Smoking Aid - 6 mins, 18 puffs (4 sec inhalations)	Not measured	SBP<br>DBP	Not reported	No
Moheimani et al, 2017 [34]	Open label, randomized crossover study	N = 33 Nicotine naïve participants	N = 33 ECN<br><br>N = 33 EC0<br><br>N = 33 Sham	N = 15 used EC - Greensmoke cigalike device w and w/o nicotine<br><br>N = 18 used EC - eGo-One by Joyetech, 2nd gen pen-like device w and w/o nicotine<br><br>N = 33 used sham EC w/o e-liquid	ECN = 1.2% nicotine<br><br>EC0 = 0% nicotine<br><br>Sham = no e-liquid	First 6 participants - 10 mins, with a puff every 30 sec with 3 sec inhalations<br><br>Last 27 participants - 30 mins, 60 puffs every 30 sec with 3 sec inhalations	Plasma nicotine<br><br>Plasma cotinine	SBP<br>DBP<br>HRV<br>HR	Not reported	No

Table 2 Study Characteristics: Acute Autonomic Cardiovascular Effects of ECs with Nicotine vs ECs without Nicotine

Abbreviations:
CV = cardiovascular
CVO = cardiovascular outcome
DBP=diastolic blood pressure
EC - electronic cigarette
ECN - electronic cigarette with nicotine
EC0 - electronic cigarette without nicotine
HR - heart rate
SBP = systolic blood pressure
TC - tobacco cigarette

Table 3 Study Characteristics: Chronic Autonomic Cardiovascular Effects of Electronic Cigarettes

Author & Year	Study Design	Sample Size	Exposure	Exposure Device (Device Type)	Nicotine Concentration	Duration Of Exposure	Nicotine/Cotinine Levels	Autonomic CV Outcomes	CVO Primary Outcomes	Conflict of Interest
D'Ruiz et al, 2017 [11]	5 day randomized open-label controlled trial while participants were clinically confined	N = 105 TC smokers	N = 15 Tobacco Rechargeable<br><br>N = 15 Cherry Rechargeable<br><br>N = 15 Cherry Disposable<br><br>N = 15 TC + Tobacco Rechargeable<br><br>N = 15 TC + Cherry Rechargeable<br><br>N = 15 TC + Cherry Disposable<br><br>N = 15 Nicotine Cessation	Tobacco Rechargeable blu EC Cherry<br><br>Rechargeable blu EC Cherry Disposable blu EC<br><br>Commercially available TC + Tobacco Rechargeable blu EC<br><br>Commercially available TC + Cherry Rechargeable blu EC<br><br>Commercially available TC + Cherry Disposable blu EC<br>Nicotine Cessation	Tobacco Rechargeable blu EC = 86mg of nicotine in 5 days<br><br>Cherry Rechargeable blu EC = 99mg of nicotine in 5 days<br><br>Cherry Disposable blue EC = 99mg of nicotine in 5 days<br><br>Commercially available TC + Tobacco Rechargeable blu EC = 107mg of nicotine in 5 days<br><br>Commercially available TC + Cherry Rechargeable blu EC = 80mg of nicotine in 5 days<br><br>Commercially available TC + Cherry Disposable blu EC = 89mg of nicotine in 5 days	Puff behavior and use topography were not reported.<br><br>EC's were weighed before and after use.	Not reported	SBP<br>DBP<br>HR	Not reported	Yes
Ikonomidis et al, 2018 [25]	1-month randomized crossover clinical trial	N = 70 TC smokers	N = 70 all TC smokers switched to ECN<br><br>N = 20 TC smoker controls	TC - did not say brand, conventional cigarette<br><br>ECN - eGo Epsilon BDC 1100, Nobacco TM	TC = not reported<br><br>ECN = 12 mg/mL nicotine cartridge<br><br>EC0 = 0 mg/ml	TC - 1 conventional cigarette<br><br>ECN - 7 mins<br><br>EC0 - 7 mins	Not measured	Central SBP<br>Central DBP<br>Peripheral SBP<br>Peripheral DBP<br>HR	Not reported	No

				EC0 - eGo Epsilon BDC 1100, Nobacco TM	nicotine cartridge					
Farsalinos et al, 2016 [14]	Post hoc analysis of 12-month prospective randomized controlled trial	N = 300 TC smokers	N = 100 - ECN<br><br>N = 100 – ECN<br><br>N = 100 - EC0	Rechargeable Categoria EC model 401	N = 100 - EC 2.4% nicotine<br><br>N = 100 - EC 2.4% nicotine + EC 1.8% nicotine<br><br>N = 100 - EC 0% nicotine	ad libitum (but up to a maximum of four cartridges per day	Not measured	SBP<br>DBP<br>HR	Not primary or secondary outcome	Yes
Moheimani et al, 2017 [35]	Cross sectional case control study	N = 42 (23 EC vapers and 19 nicotine naïve)	N/A	Participant chosen commercially available EC	N= 21 own EC brand	Not reported	Plasma nicotine and cotinine	HRV<br>HR<br>BP	HRV Primary outcome	No
Polosa et al, 2017 [38]	3.5 yr Prospective observational study	N = total 21 (9 EC vapers and 12 nicotine naïve)	N/A	Assortment of refillable EGO style products and more advanced refillable devices including Provari, Innokin, Joyetech eVIC, Avatar Puff	6 of the 9 EC users used EC with nicotine<br>3 of the 9 EC users used EC without nicotine	ad libitum daily use	Not measured	SBP<br>DBP<br>HR	Not reported	Yes
Veldheer et al, 2019 [45]	3mo Randomized controlled trial	N = 263 TC smokers	N = 72 Cigarette substitute users<br><br>N = 191 ECN users in one of 3 EC study arms	EC - 3.3–4.1 V, 1100 mAh rechargeable battery and a 1.5 Ohm, dual-coil, 510-style cartomizer (SmokTech, Shenzhen, China). Cigarette substitute - QuitSmart, Inc., NC. Plastic tube that resembles a cigarette	Cig-Sub arm 1 = 0mg/ml of nicotine<br><br>EC arm 2 = 0mg/ml of nicotine<br><br>EC arm 3 = 8mg/ml of nicotine<br><br>EC arm 4 = 36mg/ml of nicotine	Puff behavior and use topography were not reported, although investigators said participants were asked to track number of puffs.	Not measured	SBP<br>DBP<br>HR	Not reported	No

George et al, 2019 [18]	Prospective randomized trial	N = 145 TC smokers	N = 40 TC Smokers<br><br>N = 37 ECN users<br><br>N = 37 EC0 users	TC – commercially available<br><br>EC - Vapourlites Starter Kit<br><br>EC0 - Vapourlites Starter Kit	TC – commercially available<br><br>EC - Vapourlites Starter Kit with XR5 16 mg nicotine cartomizer<br><br>EC0 - Vapourlites Starter Kit with 0 mg nicotine cartomizer	Puff behavior and use topography were not reported, although investigators said participants were asked to track number of puffs.	Not Measured	SBP<br>DBP<br>HR	Secondary outcomes	No
Polosa et al, 2016 [39]	Retrospective study	N = 89 TC smokers	N/A	N/A	N/A	N/A	N/A	SBP<br>DBP	Primary Outcome	No

Abbreviations:
CV = cardiovascular
CVO = cardiovascular outcome
DBP=diastolic blood pressure
EC - electronic cigarette
ECN - electronic cigarette with nicotine
EC0 - electronic cigarette without nicotine
HR - heart rate
SBP = systolic blood pressure
TC - tobacco cigarette
